# Management of intermediate high-risk pulmonary embolism: a single-center retrospective study

**DOI:** 10.3389/fcvm.2025.1483968

**Published:** 2025-08-29

**Authors:** Fortune O. Alabi, Ashkan Ghaneie, Ibrahim Koury, Hadaya A. Alkhateeb, Jieying Liu, Mengni Guo, Baha Aldeen B. Fawwaz, Arelis Cordero, Fred Umeh

**Affiliations:** ^1^ Florida Lung Asthma and Sleep Specialists, Kissimmee, FL, United States; ^2^ AdventHealth- Radiology, Orlando, FL, United States; ^3^ Philadelphia College of Osteopathic Medicine- Georgia Campus, Suwanee, GA, United States; ^4^ East Tennessee State University College of Medicine- Hematology Oncology, Mountain Home, TN, United States; ^5^ Loma Linda School of Medicine- Hematology Oncology, Loma Linda, CA, United States; ^6^ AdventHealth- Internal Medicine, Orlando, FL, United States

**Keywords:** pulmonary embolism, intermediate pulmonary embolism, systemic thrombolysis, anticoagulation therapy, systemic reperfusion therapy

## Abstract

**Introduction:**

Approximately 25% of PE cases are intermediate-risk, with mortality rates between 5% and 25%. Management strategies for intermediate-risk PE remain inconsistent. This study compares clinical outcomes of intermediate high-risk PE patients receiving anticoagulation therapy alone (ACT) vs. those receiving systemic reperfusion therapy (RT) plus anticoagulation.

**Methods:**

This retrospective study at AdventHealth hospitals in Central Florida included adult PE patients with right ventricular strain diagnosed from January 1, 2019, to December 31, 2020. Exclusions were patients needing vasopressors or invasive ventilatory support at admission and those treated primarily with mechanical thrombectomy or catheter-directed thrombolytic therapy. Patients were divided into two groups: standard ACT and systemic RT plus ACT. Demographics, comorbidities, lab values, treatments, and outcomes were collected and compared.

**Results:**

Of 641 patients, 461 met inclusion criteria, with a median age of 65 and median BMI of 31.2. The cohort included 216 high-risk intermediate PE patients, with 52 patients receiving the thrombolytic therapy and 164 receiving the anticoagulation therapy. There was no significant difference in in-hospital (*p* = 0.450) or 30-day mortality rates (*p* = 0.591) between the two treatment groups. Secondary outcomes, including major bleeding (*p* = 0.569), use of vasopressors (*p* = 0.969), mechanical ventilation (*p* = 0.804), CPR (*p* = 0.450), or transfusion, also showed no significant difference. Notably, 23.2% of patients in the anticoagulation-only group required secondary thrombolytic therapy within 24 hours. Patients receiving systemic RT were younger (*p* = 0.017) and had a higher BMI (*p* = 0.012).

**Discussion:**

This study supports guidelines advising against routine use of RT for intermediate high-risk PE unless as rescue therapy. No mortality rate reduction or secondary outcome benefits were observed, highlighting the need for consistent management protocols and further research on therapeutic approaches for intermediate high-risk PE.

## Highlights

• This study supports current guidelines advising against the routine use of reperfusion therapy (RT) for intermediate high-risk pulmonary embolism (PE) unless it is used as rescue therapy.
• There was no observed decrease in mortality rates or reduction the incidence of secondary clinical outcomes for those receiving systemic RT.
• The risk of major bleeding was the same between those receiving standard anticoagulation therapy (ACT) and those receiving systemic RT.

## Introduction

Venous thromboembolism (VTE), which includes deep vein thrombosis (DVT) and pulmonary embolism (PE), affects approximately 900,000 people annually in the USA, and this number is expected to double by 2050. The incidence increases with age, and PE is considered the third most prevalent cause of cardiovascular death, accounting for 60,000–100,000 deaths per year (1, 2). Most cases of PE are considered stable with a relatively low mortality rate; however, <5% of PEs are considered massive, which has a mortality rate of up to 65%. The third group is intermediate-risk PEs, accounting for approximately 25% of PEs. It is imperative to identify patients with intermediate-risk PE among normotensive patients diagnosed with PE since it is associated with a high risk for PE-related complications. The population of patients with intermediate PE is heterogenous, with mortality rates ranging from 5 to 25% (3, 4). This group of intermediate PEs can be further subclassified into high- and low-risk groups based on risk stratification, which depends on the pulmonary embolism severity index (PESI) classification and the presence of elevated cardiac biomarkers and right ventricular strain (RVS) on transthoracic echocardiogram (TTE) or computed tomography pulmonary angiogram (CTPA).

Although the strategies for managing stable low-risk PE and massive PE are clearly defined and endorsed in many guidelines (5–7), there is no standardized treatment approach for intermediate PE. In the absence of consistent recommendations to guide the management of patients with intermediate PE, its treatments vary across institutions. Even within the same institution, it can vary from one campus to another. Standard anticoagulation therapy or reperfusion therapy is currently being used for treating patients with intermediate PE.

Due to the aforementioned adverse outcomes of intermediate-risk PE and its high risk of mortality, many studies have explored the benefits of treating intermediate PE with reperfusion therapy. Most of the studies did not reveal whether the risk of mortality was higher with thrombolytics or with heparin; however, some revealed that the group treated with only heparin required treatment escalation compared with the group treated with thrombolytics despite a similar degree of bleeding (5). Other studies reported less hemodynamic decompensation in patients who received reperfusion therapy despite increased intracranial bleeding (6). A meta-analysis performed by Nakamura et al. concluded that adjunctive reperfusion therapy does not significantly reduce the risk of mortality or recurrent PE in patients with acute intermediate PE; however, it prevents clinical deterioration that necessitates treatment escalation in patients with acute intermediate PE (7).

In this retrospective study, we compared the clinical outcomes of intermediate high-risk PE patients based on whether they received reperfusion therapy or anticoagulation therapy only.

The primary objective of this study was to compare the clinical outcomes of patients with intermediate high-risk PE who received only anticoagulation therapy to those who received systemic reperfusion therapy and anticoagulation therapy. The secondary endpoint was 30-day mortality of patients with intermediate high-risk PE who received only anticoagulation therapy compared with those who received anticoagulation and systemic reperfusion therapy.

## Study design and methods

This was a single-center retrospective study performed at AdventHealth hospitals in Central Florida. Adult patients aged 18 years or older with a diagnosis of PE with RVS who were admitted between January 1, 2019, and December 31, 2020 were considered eligible for inclusion in the study. However, only patients who met the radiographic definition of RVS with at least an RV/LV ratio ≥1.0 were included in the study. Patients with PE requiring vasopressor agents or invasive ventilatory support on admission were excluded. We also excluded patients who were primarily treated with mechanical thrombectomy or with catheter-directed thrombolytics.

Radiologists on the research team reviewed the chest CTPA scans of patients to ensure that all the included patients met the radiographic criteria for RVS, which consisted of the following: condition A and either condition B or C.

(A) RV/LV ratio ≥1.0 measured on anatomic axial images
(B) Flattening of the interventricular septum
(C) Reflux into the inferior vena cava (IVC)

**Figure 1:**
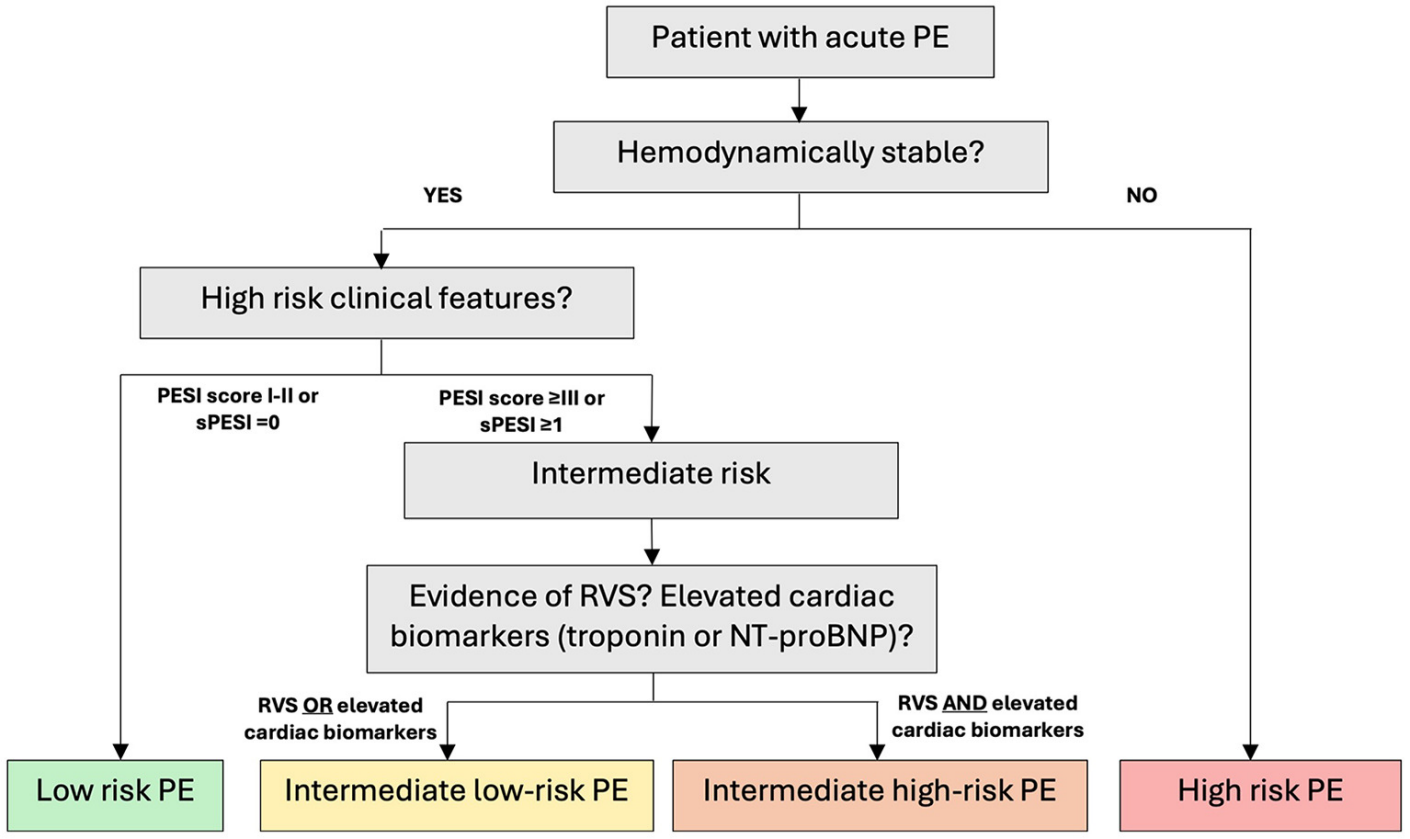
Pe risk stratification (ESC 2019). ESC, European Society of Cardiology; PE, pulmonary embolism; RVS, right ventricular strain; PESI, pulmonary embolism severity index; sPESI, simplified pulmonary embolism severity index; NT-proBNP, N-terminal pro b-type natriuretic peptide.

The definition of intermediate high-risk PE was based on the 2019 ESC guidelines (8) (Figure 1): a pulmonary embolism severity index (PESI) score ≥III or simplified pulmonary embolism severity index (sPESI) score ≥1, RVS on CTPA, and elevated cardiac biomarkers [troponin T or N-terminal pro b-type natriuretic peptide (NT-proBNP)]. While not all patients had both biomarkers measured, all patients included in the intermediate high-risk PE group had at least once elevated cardiac biomarker prior to treatment, consistent with ESC classification. The intermediate low-risk PE group was identified by a PESI score ≥III or sPESI score ≥1, and the presence of RVS without elevated cardiac biomarkers. The patients were divided into two groups: those who received standard anticoagulation therapy (unfractionated heparin [UFH] or low molecular weight heparin [LMWH]) and those who received systemic reperfusion therapy in addition to standard anticoagulation therapy.

### Data collection

The following data were collected from the patients’ charts:

Demographic data: age, sex and BMI.

Comorbidities: chronic renal insufficiency, chronic liver disease, chronic pulmonary disease, history of previous VTE, history of malignancy.

Cardiovascular risk factors: systemic hypertension, congestive heart failure (CHF), coronary artery disease (CAD).

Laboratory values: hemoglobin (Hgb) on admission, on discharge, and lowest during admission; platelet count on admission; creatinine/BUN on admission; PT/INR, APTT on admission; COVID-19 positive, negative, or unknown; cardiac biomarkers (Troponin, NT-proBNP).

Transfusion: Yes/No.

The types of treatment received were as follows: Standard anticoagulation therapy (ACT) (LMWH or UFH), primary systemic reperfusion therapy (RT), or secondary systemic RT. Primary systemic RT was defined as administration of systemic thrombolysis with tissue plasminogen activator (tPA) in addition to standard ACT. Secondary systemic RT, or emergency RT, was defined as any escalation to systemic thrombolysis with tPA, catheter-directed thrombolysis, or mechanical thrombectomy after treatment with either standard ACT or primary RT were trialed and failed after 24 hours. This outcome was included to capture early treatment failure with anticoagulation alone and the clinical need for urgent intervention.

Risk stratification scores: PESI or sPESI; TTE findings (RVS); venous Doppler findings

The clinical outcomes of interest are as follows:

 • Major bleeding (as defined according to the International Society on Thrombosis and Hemostasis criteria)
  1. Fatal bleeding.
  2. Symptomatic bleeding in a critical area or organ, such as intracranial, intraspinal, intraocular, retroperitoneal, intra-articular or pericardial, or intramuscular with compartment syndrome.
  3. Bleeding decreasing the Hgb level by 2 g/dl (1.24 mmol/L) or more or that leads to the transfusion of two or more units of whole blood or red blood cells.
 • Use of vasopressors after 12 hours of therapy
 • Need for mechanical ventilator assistance (invasive or noninvasive) following 12 hours of therapy
 • CPR during hospitalization
 • In-hospital mortality
 • 30-day mortality

### Statistical analysis plan

The data were imported into and analyzed using SPSS version 23 for Windows (IBM Corp., Armonk, NY). Frequency and descriptive statistics were used to describe the demographic characteristics of the sample. The same statistics were used to generate measures of prevalence rates in the two groups (standard ACT vs. thrombolytics) for the primary and secondary outcomes. Chi-square tests of independence were used to compare the composite primary outcome (in-hospital mortality, secondary [emergency] thrombolytic treatment, need for catecholamine support of blood pressure in patients who had been treated for more than 12 hours, need for endotracheal intubation, cardiopulmonary resuscitation, and/or bleeding) and the secondary outcome of 30-day mortality between the treatment groups. The frequency, percentage, and unadjusted odds ratio (OR) with 95% confidence intervals (95% CI) were reported and interpreted for the chi-square analyses. As the data for continuous demographic and clinical parameters (age, BMI, Hgb, creatinine, PT/INR, platelets, and risk stratification scores) were not normally distributed, medians and interquartile ranges (IQRs) were reported to summarize these variables. Mann‒Whitney *U*-tests were used to compare these variables between the treatment groups. Statistical significance was assumed at an alpha value of 0.05. Multiple comparison corrections were not applied due to the exploratory nature of this early-stage research.

### Ethical considerations

The AdventHealth IRB approved this study, and the need for consent was waived because of the retrospective nature of the study. There was no more than minimal risk to the study participants.

## Results

### Results for all patients with intermediate PE (low- and high-risk patients)

**Figure 2:**
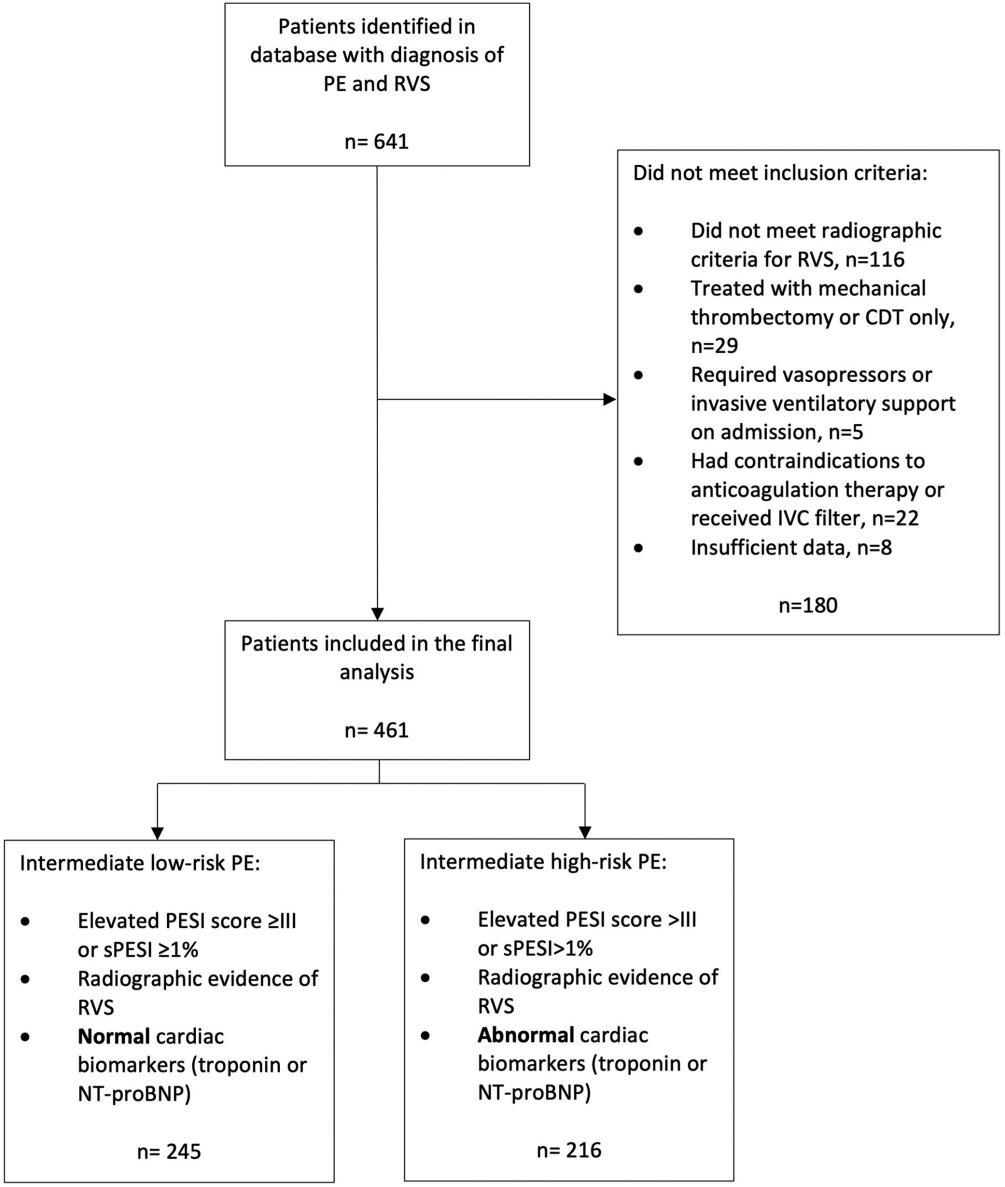
Inclusion and exclusion criteria. PE, pulmonary embolism; RVS, right ventricular strain; CDT, catheter-directed thrombectomy; IVC, inferior vena cava; PESI, pulmonary embolism severity index; sPESI, simplified pulmonary embolism severity index; NT-proBNP, N-terminal pro b-type natriuretic peptide.

In total, 641 patients with PE associated with RVS were identified from the database of radiology departments between January 1, 2019, and December 31, 2020. A total of 525 patients met the radiographic criteria for RVS after a thorough review of the CTPA images by the attending radiologists (AG and IK) involved in the study. Overall, twenty-two patients were excluded because they were not candidates for ACT or had an IVC placed, twenty-nine patients were excluded because they were treated with either catheter-directed thrombolytic therapy or mechanical thrombectomy, and five patients were excluded because they were treated with vasopressors or required invasive ventilatory support. The data of 461 subjects with intermediate PE were retrieved after excluding the eight patients who did not have data for the study measures (e.g., outcomes, demographics, cardiovascular risk factors, mean laboratory values, and risk stratification scores) from further data analysis (Figure 2).

Table 1 shows the demographics of the study subjects. The median age was 65.0 years (IQR = 22), and the median BMI was 31.2 (IQR = 11). Approximately half of the subjects were male (52.1%). Approximately one-third of the subjects had abnormal troponin levels on admission (36.9%) and abnormal NT-proBNP levels on admission (36.4%).

**Table 1:** Demographics of intermediate PE, both low-risk and high-risk.

Demographics of Intermediate PE, both low-risk and high-risk		
	*n*	%
Age, median (IQR)	65.0 (22)	
BMI, median (IQR)	31.2 (11)	
Sex		
Female	221	47.9
Male	240	52.1
Troponin on admission		
Abnormal	170	36.9
Normal	247	53.6
Missing	44	9.5
NT-proBNP on admission		
Abnormal	168	36.4
Normal	189	41.0
Missing	104	22.6
History of malignancy		
Yes	79	17.1
No	382	82.9
Chronic renal insufficiency		
Yes	22	4.8
No	439	95.2
Chronic liver disease		
Yes	10	2.2
No	451	97.8
Chronic pulmonary disease		
Yes	86	18.7
No	375	81.3
History of previous VTE		
Yes	90	19.5
No	371	80.5
Treatment		
Thrombolytic therapy (TT)	74	16.1
Anticoagulation therapy (ACT)	387	83.9

Sample size, *N* = 461. *N* = 459 for BMI.

### Results for patients with high-risk intermediate PE

A high-risk intermediate PE patient was defined as a patient with a high sPESI score, RVS, and abnormal troponin or NT-proBNP on admission. A total of 216 subjects were identified as high-risk intermediate PE patients.

Table 2 shows the demographics of these subjects. The median age was 67 years (IQR = 20), and the median BMI was 31.5 (IQR = 11.9). Approximately half of the subjects were female (50.5%). Approximately two-thirds of the subjects had abnormal troponin levels on admission (68.5%) and abnormal NT-proBNP levels on admission (63.0%). Fewer than 20% of the subjects had a history of chronic malignancy (19.9%) or previous VTE (17.6%). Over 20% of the subjects had chronic pulmonary disease (21.8%). Less than 10% of the subjects had chronic renal insufficiency (6.9%) or chronic liver disease (3.7%). A total of 24.1% of the subjects received primary thrombolytic RT, and 75.9% of the subjects received ACT.

**Table 2:** Demographics of high-risk intermediate PE patients.

Demographics of High-risk Intermediate PE patients		
	*n*	%
Age, median (IQR)	67 (20)	
BMI, median (IQR)	31.5 (11.9)	
Sex		
Female	109	50.5
Male	107	49.5
Troponin on admission		
Abnormal	148	68.5
Normal	66	30.6
Missing	2	0.9
NT-proBNP on admission		
Abnormal	136	63.0
Normal	48	22.2
Missing	32	14.8
History of malignancy		
Yes	43	19.9
No	173	80.1
Chronic renal insufficiency		
Yes	15	6.9
No	201	93.1
Chronic liver disease		
Yes	8	3.7
No	208	96.3
Chronic pulmonary disease		
Yes	47	21.8
No	169	78.2
History of pervious VTE		
Yes	38	17.6
No	178	82.4
Treatment		
Thrombolytic therapy (TT)	52	24.1
Anticoagulation therapy (ACT)	164	75.9

Sample size, *N* = 216. *N* = 214 for BMI.

Table 3 presents the cross-tabulation of the study outcome measures and treatment. There was a statistically significant relationship between treatment and secondary thrombolytic therapy after 24 h (*p* < 0.001). Among the subjects who received tPA (primary RT), none received secondary thrombolytic RT after 24 h, while among the subjects who received ACT only, 23.2% received secondary thrombolytic RT after 24 h.

**Table 3:** Frequency (%) of study outcome measures by treatment.

Frequency (%) of study outcome measures by treatment						
	TT (*N* = 52)	ACT (*N* = 164)	*χ* ^2^	df	*p*	OR [95% CI]
Secondary intervention after 24 h						
Yes	0	38 (23.2)	14.621	1	< 0.001	NA
No	52 (100)	126 (76.8)				
Major bleeding						
Yes	10 (19.2)	26 (15.9)	0.324	1	0.569	1.264 [0.564, 2.832]
No	42 (80.8)	138 (84.1)				
Use of vasopressor after 12 h of therapy						
Yes	9 (17.3)	28 (17.1)	0.002	1	0.969	1.017 [0.445, 2.231]
No	43 (82.7)	136 (82.9)				
Need mechanical ventilator assist after 12 h of therapy						
Yes	9 (17.3)	26 (15.9)	0.061	1	0.804	1.111 [0.484, 2.552]
No	43 (82.7)	138 (84.1)				
CPR during hospitalization						
Yes	7 (13.5)	16 (9.8)	0.570	1	0.450	1.439 [0.557, 3.716]
No	45 (86.5)	148 (90.2)				
In-hospital mortality						
Yes	7 (13.5)	16 (9.8)	0.570	1	0.450	1.439 [0.557, 3.716]
No	45 (86.5)	148 (90.2)				
30-day mortality						
Yes	7 (16.3)	18 (13.0)	0.288	1	0.591	1.296 [0.502, 3.349]
No	36 (83.7)	120 (87.0)				
Unknown	9	26				

TT, Thrombolytic therapy; ACT, Anticoagulation therapy. For 30-day mortality, the chi-square test was only performed using the “Yes” and “No” categories. NA, not available, as the OR values could not be calculated due to zero events.

There was no statistically significant relationship between treatment and major bleeding (*p* = 0.569), the use of vasopressors after 12 h of therapy (*p* = 0.969), the need for mechanical ventilator assistance after 12 h of therapy (*p* = 0.804), CPR during hospitalization (*p* = 0.450), in-hospital mortality (*p* = 0.450), or 30-day mortality (*p* = 0.591). Table 4 presents the cross-tabulation of categorical demographic variables and treatment. There was no statistically significant relationship between treatment and sex (*p* = 0.939), troponin level at admission (*p* = 0.740), BNP level at admission (*p* = 0.931), history of malignancy (*p* = 0.590), chronic renal insufficiency (*p* = 0.313), chronic liver disease (*p* = 0.105), chronic pulmonary disease (*p* = 0.612), or history of previous VTE (*p* = 0.631).

**Table 4:** Frequency (%) of categorical demographic variables by treatment.

Frequency (%) of categorical demographic variables by treatment						
	TT (*N* = 52)	ACT (*N* = 164)	χ^2^	df	*p*	OR [95% CI]
Sex						
Female	26 (50.0)	83 (50.6)	0.006	1	0.939	0.976 [0.523, 1.821]
Male	26 (50.0)	81 (49.4)				
Troponin on admission						
Abnormal	35 (67.3)	113 (69.8)	0.110	1	0.740	0.893 [0.457, 1.744]
Normal	17 (32.7)	49 (30.2)				
NT-proBNP on admission						
Abnormal	32 (74.4)	104 (73.8)	0.007	1	0.931	1.035 [0.474, 2.260]
Normal	11 (25.6)	37 (26.2)				
History of malignancy						
Yes	9 (17.3)	34 (20.7)	0.290	1	0.590	0.800 [0.355, 1.802]
No	43 (82.7)	130 (79.3)				
Chronic renal insufficiency						
Yes	2 (3.8)	13 (7.9)	1.017	1	0.313	0.465 [0.101, 2.130]
No	50 (96.2)	151 (92.1)				
Chronic liver disease						
Yes	0	8 (4.9)	2.634	1	0.105	NA
No	52 (100)	156 (95.1)				
Chronic pulmonary disease						
Yes	10 (19.2)	37 (22.6)	0.257	1	0.612	0.817 [0.374, 1.784]
No	42 (80.8)	127 (77.4)				
History of pervious VTE						
Yes	8 (15.4)	30 (18.3)	0.230	1	0.631	0.812 [0.347, 1.902]
No	44 (84.6)	134 (81.7)				

TT, Thrombolytic therapy; ACT, Anticoagulation therapy; VTE, venous thromboembolism. Missing values were not included in the chi-square analysis for troponin on admission or NT-proBNP on admission. NA, not available, as the OR values could not be calculated due to zero events.

Table 5 presents descriptive statistics of continuous demographic variables (age and BMI) by treatment. Age and BMI were not normally distributed according to the results of the Shapiro‒Wilk test (*p* < 0.05). Hence, the median and IQR were used to summarize age and BMI. Subjects who received tPA (median = 63, IQR = 17) were significantly younger than subjects who received ACT (median = 69, IQR = 22) (*p* = 0.017). The BMI of subjects who received tPA (median = 33.7, IQR = 12.9) was significantly greater than that of subjects who received ACT (median = 31.0, IQR = 11.9) (*p* = 0.012).

**Table 5:** Descriptive statistics of continuous demographic variables by treatment.

Descriptive statistics of continuous demographic variables by treatment									
	Median (IQR)		Shapiro‒Wilk test		Mann‒Whitney *U*-test				
	TT	ACT	TT	ACT	U	SE	z	*p*	r
Age	63 (17)	69 (22)	W = 0.931, df = 52, *p* = 0.005	W = 0.976, df = 164, *p* = 0.005	5,200	392.577	2.384	0.017	0.162
BMI	33.7 (12.9)	31.0 (11.9)	W = 0.941, df = 51, *p* = 0.014	W = 0.911, df = 163, *p* < 0.001	3,182	385.901	−2.525	0.012	0.173

TT, thrombolytic therapy; ACT, anticoagulation therapy. For age, *N* = 52 for TT, *N* = 164 for ACT. For BMI, *N* = 51 for TT, *N* = 163 for ACT.

Table 6 presents the cross-tabulation of cardiopulmonary risk factors and treatment. There was no statistically significant relationship between treatment and any of the comorbidities, such as systemic hypertension (*p* = 0.188), congestive heart failure (*p* = 0.083), and coronary artery disease (*p* = 0.092).

**Table 6:** Frequency (%) of cardiovascular risk factors by treatment.

Frequency (%) of cardiovascular risk factors by treatment						
	TT (*N* = 52)	ACT (*N* = 164)	χ^2^	df	*p*	OR [95% CI]
Systemic hypertension						
Yes	27 (51.9)	102 (62.2)	1.732	1	0.188	0.656 [0.350, 1.231]
No	25 (48.1)	62 (37.8)				
CHF						
Yes	1 (1.9)	15 (9.1)	3.003	1	0.083	0.195 [0.025, 1.512]
No	51 (98.1)	149 (90.9)				
CAD						
Yes	3 (5.8)	24 (14.6)	2.837	1	0.092	0.357 [0.103, 1.239]
No	49 (94.2)	140 (85.4)				

TT, thrombolytic therapy; ACT, anticoagulation therapy; CAD, coronary artery disease; CHF, congestive heart failure.

Table 7 presents descriptive statistics of the mean laboratory values by treatment. Hgb on admission for subjects who received tPA (median = 14.2, IQR = 3.6) was significantly greater than Hbg on admission for subjects who received ACT (median = 13.0, IQR = 3.5) *p* = 0.031). There was no statistically significant difference in the lowest Hbg at admission (*p* = 0.373), platelet count (*p* = 0.563), or coagulation profile, such as, creatinine (*p* = 0.287), blood urea nitrogen (*p* = 0.083), international normalized ratio (*p* = 0.287), and activated partial thromboplastin time (*p* = 0.152).

**Table 7:** Descriptive statistics of mean laboratory values by treatment.

Descriptive statistics of mean laboratory values by treatment									
	Median (IQR)		Shapiro‒Wilk test		Mann‒Whitney *U*-test				
	TT	ACT	TT	ACT	U	SE	z	*p*	r
Hgb on admission	14.2 (3.6)	13.0 (3.5)	W = 0.953, df = 52, *p* = 0.040	W = 0.978, df = 164, *p* = 0.009	3,416	392.646	−2.160	0.031	0.147
Lowest Hgb during admission	11.3 (3.3)	10.5 (4.4)	W = 0.943, df = 52, *p* = 0.015	W = 0.973, df = 164, *p* = 0.003	3,914.5	392.649	−0.890	0.373	0.061
Plt count on admission	226.5 (88)	211.0 (108)	W = 0.943, df = 52, *p* = 0.015	W = 0.984, df = 164, *p* = 0.052	4,037	392.687	−0.578	0.563	0.039
Cr on admission	1.1 (0.5)	1.0 (0.5)	W = 0.928, df = 52, *p* = 0.004	W = 0.351, df = 164, *p* < 0.001	3,846	392.636	−1.065	0.287	0.072
BUN on admission	15.5 (9)	18.0 (11)	W = 0.850, df = 52, *p* < 0.001	W = 0.729, df = 164, *p* < 0.001	4,945	392.278	1.736	0.083	0.118
INR on admission	1.1 (0.2)	1.1 (0.2)	W = 0.259, df = 52, *p* < 0.001	W = 0.697, df = 163, *p* < 0.001	4,654	390.314	1.066	0.287	0.073
aPTT on admission	29.4 (5.1)	30.4 (8.6)	W = 0.371, df = 52, *p* < 0.001	W = 0.452, df = 161, *p* < 0.001	4,739	386.364	1.431	0.152	0.098

TT, thrombolytic therapy; ACT, anticoagulation therapy; Hgb, hemoglobin; plt, platelet; Cr, creatinine; BUN, blood urea nitrogen; INR, international normalized ratio; aPTT, activated partial thromboplastin time. *N* = 52 for TT, *N* = for ACT. One missing value for the INR at admission and 3 missing values for aPTT at admission.

Table 8 presents the cross-tabulation of COVID-19 and treatment. There was no statistically significant relationship between treatment and COVID-19 (*p* = 0.459). The prevalence of COVID-19 was approximately 10% for each treatment group (9.1% for tPA and 9.1% for ACT). Table 9 shows that there was no statistically significant relationship between treatment and RV strain on ECHO (*p* = 0.332) or DVT on venous Doppler US (*p* = 0.099). The prevalence of the RV strain on echocardiography was 56.9% (tPA) and 49.0% (ACT). The prevalence of DVT on venous Doppler US was 70.5% (tPA) and 56.6% (ACT).

**Table 8:** Frequency (%) of COVID-19 positivity by treatment.

Frequency (%) of COVID-19 positivity by treatment						
	TT (*N* = 52)	ACT (*N* = 164)	χ^2^	df	*p*	OR [95% CI]
COVID-19						
Negative	19 (95.0)	60 (89.6)	0.547	1	0.459	2.217 [0.256, 19.180]
Positive	1 (5.0)	7 (10.4)				
Unknown	32	97				

TT, Thrombolytic therapy; ACT, Anticoagulation therapy. The chi-square test was only performed using the “negative” and “positive” categories.

**Table 9:** Frequency (%) of risk stratification scores by treatment.

Frequency (%) of risk stratification scores by treatment						
	TT (*N* = 52)	ACT (*N* = 164)	χ^2^	df	*p*	OR [95% CI]
RV strain on ECHO						
Yes	29 (56.9)	77 (49.0)	0.942	1	0.332	1.370 [0.725, 2.588]
No	22 (43.1)	80 (51.0)				
Unknown	1	6				
DVT on V. Doppler US						
Yes	31 (70.5)	82 (56.6)	2.714	1	0.099	1.832 [0.886, 3.787]
No	13 (29.5)	63 (43.4)				
Unknown	8	19				

TT, Thrombolytic therapy; ACT, Anticoagulation therapy; V. Doppler US, venous doppler ultrasound. The chi-square test was only performed using the “Yes” and “No” categories for RV strain on ECHO and DVT on V. Doppler US.

## Discussion

The population of patients with intermediate PE is heterogeneous comprising patients with low and high-risk PE based on the European Society of Cardiology (ESC) risk stratification. According to the 2019 ESC guidelines, patients at intermediate–high risk are classified as having PESI class III or higher or sPESI class >1 and the presence of at least one cardiac biomarker plus imaging signs of RVS. This retrospective study was conducted primarily to compare the clinical outcomes of patients with intermediate high-risk PE treated based on the recommendations of the 2019 ESC guidelines with those of patients not treated in accordance with the guidelines.

The management of intermediate-risk PE has been a matter of contention for years. According to most guidelines, thrombolysis is not recommended, except as a rescue therapy in patients whose conditions deteriorate despite treatment with ACT. According to the 2019 ESC guidelines, RT is not recommended for intermediate-risk PE (8). Furthermore, RT is not recommended for patients with PE without hypotension according to the updated CHEST guidelines put forward in 2021 (9). Epidemiologic studies have confirmed that the rate of mortality due to VTE continues to decrease as a result of treatment with ATC. The clinical data of 23,858 patients with acute PE registered in a large international registry between 2001 and 2013 were reviewed. The length of stay (LOS) decreased from 13.6 days to 9.3 days, and there was a reduction in short-term all-cause and PE-specific mortality rates. The use of RT increased from 0.7% to 1.0% during that period (10). In another study from Germany conducted by Keller et al. in 2019, researchers analyzed the characteristics, comorbidities, treatment, and in-hospital outcomes of 885,806 patients with PE enrolled between 2005 and 2015. Similarly, the LOS decreased from 12 days to 8 days, and the in-hospital mortality rate decreased from 20.4% to 13.9%. Systemic thrombolytics were administered to 4.2% of the patients in the study, 50.9% of whom were hemodynamically stable. The in-hospital mortality rate was 76.6% in patients who presented with hemodynamic instability and 10.8% in hemodynamically stable patients. The case fatality rate among hemodynamically unstable patients 28.6% for those who received RT and 49.9% for those who did not receive reperfusion therapy. However, the inclusion of all hospitalized patients with PE (hemodynamically stable and unstable) in the analysis revealed that those who underwent thrombolysis had a higher in-hospital mortality rate than did those who did not receive RT (44.7% vs. 15.4%, *p* < 0.001) (11).

Although the study conducted in Germany provided valuable information on the use of reperfusion therapy in patients who were hemodynamically unstable in real-world practice, which in essence was the focus of their study, limited information was provided about the outcomes of reperfusion therapy in 2.12% (18,784/885,806) of their cohort that received thrombolytics despite being hemodynamically stable. Our study was performed primarily to evaluate the clinical outcomes of reperfusion therapy in hemodynamically stable patients with (intermediate high-risk) PE and to provide additional evidence for or against the recommendation of the 2019 ESC guidelines.

The radiographic criteria for RVS in this study were very stringent. The presence of an RV/LV ratio ≥1 plus at least either interventricular septum flattening or contrast reflux into the IVC is needed. The identification of RV strain in this study was based on radiographic criteria, with only approximately 50% of the patients showing echocardiographic evidence of RV strain. Radiographic evidence of RVS is used in most centers because of the ease and timeliness of obtaining this information from CTPA. Radiographic signs of RVS have also been shown to be sensitive but not as specific as echocardiographic signs (12). The absence of RVS on CTA of the chest can be used to exclude RV dysfunction in patients with PE (13). Likewise, the absence of RVS on CTA of the chest is sufficient in most cases to exclude RV dysfunction in patients with PE (13).

The CTPA quantification of RVS is an accurate predictor of in-hospital death related to PE (14, 15). George et al.'s study showed that TTE and CTA had the same predictive ability, with similar areas under the curve (AUCs = 0.81 vs. 0.80). Both the CT RV/LV diameter ratio and the TTE RVS were independently associated with PE-related 30-day mortality (16).

This study did not show any benefits of RT for patients with intermediate high-risk PE based on RVS on CTPA of the chest. There was also no significant relationship between treatment and the presence of RVS on echocardiograms. There was no significant difference in in-hospital or 30-day mortality rate between the patients who received RT and those who received ACT. There was no significant difference in any of the secondary clinical outcome endpoints between the two groups. While the mortality outcomes showed no difference between groups, it is noteworthy that nearly one-quarter of patients who initially received anticoagulation therapy alone required escalation to reperfusion therapy within 24 hours. This outcome was included as a proxy for early clinical deterioration and treatment failure. Although not a standard endpoint in PE trials, it is relevant in clinical practice and suggests that a subset of patients classified as intermediate high-risk may benefit from earlier identification and closer monitoring for signs of decompensation.

Surprisingly, the risk of major bleeding was the same between the two groups. This may be partially because 23% of the patients in the ATC group required secondary thrombolytic agents. However, in half of the patients, mechanical thrombectomy or catheter-directed therapy was adopted rather than systemic thrombolytic therapy. In contrast to our findings, in the meta-analysis by Chatterjee et al., RT in patients with intermediate-risk PE was associated with lower all-cause mortality rates, greater risks of major bleeding, and intracranial hemorrhage (ICH) (17). Similarly, the meta-analysis by Riera-Mestre et al. also showed a reduction in mortality rates despite increased major bleeding and ICH (18), and our study did not show a decrease in mortality rate or an increased risk of bleeding between the two groups. The findings of these meta-analyses could be partially explained by the multiple overlapping systemic reviews and the fact that only a few studies contributed to the majority of the patients in the meta-analysis (19).

Unlike the study by Carroll et al., which revealed that the multimodality (evidence of RVS on EKG, CT, and TTE) approach for the assessment of RVS was superior for risk stratification (20), in our study, the addition of echocardiographic evidence of RVS did not lead to a difference in the clinical outcomes between the two groups. In this study, no difference was observed in the frequency of DVT between the two groups. There was also no difference between the clinical outcomes between the groups regarding the presence of DVT.

The demographics of the patients with high-risk intermediate PE were similar to those of the entire cohort with intermediate PE in terms of age and sex distributions, but the patients in the high-risk intermediate PE group were slightly younger and had a higher BMI. The median age of the population in this study was also similar to that in the PEITHO trial (6). The percentage of patients with comorbid conditions and malignancies was greater in this study than in the PEITHO trial.

Since the completion of this study, catheter-directed therapy (CDT) has become more commonly used in the treatment of sub-massive pulmonary embolism (PE). However, a definitive reduction in mortality associated with CDT has yet to be demonstrated. Some studies have reported complete resolution of pulmonary hypertension and normalization of right ventricular (RV) dilatation following CDT (21).

More recently, there has been a notable shift toward the use of mechanical thrombectomy in patients with intermediate-risk PE, particularly at AdventHealth hospital system in Orlando. This trend reflects a broader national shift in clinical practice  (22, 23). Despite the growing preference for mechanical thrombectomy, several studies have shown comparable outcomes between CDT and mechanical thrombectomy in terms of in-hospital mortality, bleeding risk, and hemodynamic parameters  (24).

Ongoing prospective trials such as HI-PEITHO (NCT04790370) and PEITHO-3 (NCT04430569) are expected to provide high-quality evidence regarding the role of systemic and catheter-based therapies in intermediate-risk PE. HI-PEITHO aims to investigate the safety and efficacy of imaging-guided thrombolysis in patients with intermediate-high risk features, while PEITHO-3 focuses on optimizing reperfusion strategies and patient selection criteria. These studies will be instrumental in validating current risk stratification models and guiding future clinical practice (25, 26).

Notably, the PEERLESS trial—a prospective, multicenter, randomized controlled study involving 550 patients with intermediate-risk PE characterized by RV dilatation and additional clinical risk factors—randomized participants 1:1 to large-bore mechanical thrombectomy (LBMT) or CDT. The trial met its primary endpoints, showing lower rates of clinical deterioration, need for bailout interventions, and postprocedural intensive care unit (ICU) utilization in the LBMT group compared to CDT. Importantly, there was no significant difference in overall mortality or major bleeding, favoring LBMT as a potentially more efficient intervention for this patient population (27).

The management of intermediate-risk PE continues to evolve, with increasing emphasis on individualized risk stratification and targeted intervention. Traditional tools such as PESI and sPESI remain valuable, but newer models incorporating advanced imaging, cardiac biomarkers, and dynamic clinical parameters are under active investigation. The emergence of catheter-based and mechanical therapies further underscores the need for refined selection criteria to balance therapeutic benefit against bleeding risk. Future guidelines will likely integrate these advancements to better tailor treatment strategies to specific risk phenotypes.

While new tools and trials continue to advance the management of PE, it is important to optimize the application of existing risk stratification methods. The underutilization of cardiac biomarker such as troponin and NT-proBNP in the management of PE is a known issue in real-world clinical settings. This is often due to a combination of system-level and clinician-level factors. From a systems perspective, emergency departments may prioritize rapid imaging (e.g., CTPA) and stabilization over comprehensive biomarker assessment, particularly in high-risk settings. Biomarker results can be delayed or overlooked if they are not automatically bundled with PE evaluation protocols. At the provider level, there may be limited awareness of how cardiac biomarkers contribute to risk stratification, especially among clinicians who are less familiar with the ESC guidelines or who rely primarily on hemodynamic parameters and radiographic findings. Some providers may also perceive biomarkers as redundant when imaging already confirms RVS. Finally, in resource-limited or community-based hospitals, logistical barriers such as lab turnaround time, cost considerations, or workflow interruptions may further hinder consistent biomarker testing. These gaps underscore the need for education, protocol standardization, and clinical decision support to improve risk stratification and adherence to guideline-recommended PE management pathways.

### Limitations

This study has many limitations because of its retrospective nature. The dataset was obtained from the electronic medical records system across many campuses of a large medical center in Central Florida. Clinical parameters (PESI or sPESI) were not reported in the medical records. The impact of this clinical parameter on the choice of therapy cannot be ascertained. There is also the element of clinical gestalt that might have informed the choice of treatment, which is impossible to capture. Patients with PE, high PESI/sPESI, and elevated cardiac biomarkers but no evidence of RVS were not included in the intermediate low-risk group in this study, which differs slightly from ESC guidelines. However, this does not impact the intermediate high-risk PE group, which is the primary focus of this study.

Although the incidence of major bleeding based on the ISTH criteria was the same between the two groups, we did not account for clinically relevant nonmajor bleeding in this study, which could have led to a difference. The incidence of major bleeding among patients who received secondary thrombolytics might also be inaccurate since several of them received mechanical thrombectomy or catheter-directed thrombolytics, both of which have a reduced risk of bleeding compared with systemic thrombolytics. We were also unable to reliably differentiate between patients who received a standard vs. reduced dose of systematic alteplase, and therefore could not assess whether dose variation impacted outcomes such as bleeding risk or escalation of care. The in-hospital and 30-day mortality rates were the same between the two groups; however, the mortality could not be directly attributed to PE in some of the patients. The absence of an association between treatment and underlying comorbidities could be secondary to underreporting of these conditions in the medical records of the patients whose charts were reviewed. Despite excluding some of the patients from the analysis because they did not meet the stringent radiographic RVS criteria used in this study, it is possible that some patients might have been missed.

This study did not include multivariate regression analysis due to the limited sample size in the systemic reperfusion therapy group (*n* = 52). Performing a multivariable model with several covariates in a small cohort would risk overfitting and reduce the reliability of estimates. Given the exploratory nature of this retrospective study, we focused on unadjusted group comparisons. This limitation highlights the need for larger prospective studies that are adequately powered to adjust for multiple confounding variables and confirm these findings.

## Conclusion

In summary, this study supports several guidelines that recommend against the use of RT in patients with intermediate high-risk PE, except for patients who are receiving rescue reperfusion therapy for those who deteriorate clinically. Although we did not observe a significant difference between the groups in terms of the risk of major bleeding, other studies have shown an increased risk of major bleeding without clear-cut evidence of benefits compared to patients receiving only ACT. There was also no difference in the in-hospital mortality rate or the 30-day mortality rate between the two groups.

## Data Availability

The raw data supporting the conclusions of this article will be made available by the authors, without undue reservation.

## Glossary

VTE: venous thromboembolism
DVT: deep vein thrombosis
PE: pulmonary embolism
TTE: transthoracic echocardiogram
CTPA: computed tomography pulmonary angiography
CDT: Catheter-directed thrombolysis
RVS: right ventricular strain
PESI: pulmonary embolism severity index
sPESI: simplified pulmonary embolism severity index
IVC: inferior vena cava
RV: right ventricle
LV: left ventricle
NTproBNP: N-terminal pro b-type natriuretic peptide
UFH: unfractionated heparin
LMWH: low molecular weight heparin
BMI: body mass index
CHF: congestive heart failure
CAD: coronary artery disease
BUN: blood urea nitrogen
PT: prothrombin time
INR: international normalized ratio
aPTT: activated partial thromboplastin time
COVID-19: coronavirus disease 2019
RT: reperfusion therapy
ACT: anticoagulation therapy
tPA: tissue plasminogen activator
CPR: cardiopulmonary resuscitation
Hgb: hemoglobin
IQR: interquartile range
SPSS: Statistical Package for the Social Sciences
BMI: body mass index
OR: Odds ratio
CI: confidence interval
US: ultrasound
ESC: European Society of Cardiology
CHEST: American College of Chest Physicians
LOS: length of stay
AUC: area under the curve
CT: computed tomography
CTA: computed tomography angiography
EKG: electrocardiogram
ICH: intracranial hemorrhage
PEITHO: Pulmonary Embolism International THrOmbolysis
ISTH: International Society on Thrombosis and Haemostasis.
